# Altered Differentiation of Tendon-Derived Stem Cells in Diabetic Conditions Mediated by Macrophage Migration Inhibitory Factor

**DOI:** 10.3390/ijms22168983

**Published:** 2021-08-20

**Authors:** Du-Hwan Kim, Sun-Up Noh, Seoung-Wan Chae, Sang-Jun Kim, Yong-Taek Lee

**Affiliations:** 1 Department of Physical Medicine and Rehabilitation, College of Medicine, Chung-Ang University, Seoul 06973, Korea; ri-pheonix@hanmail.net; 2 Medical Research Institute, Kangbuk Samsung Hospital, Sungkyunkwan University School of Medicine, Seoul 03181, Korea; nsu0919@naver.com; 3 Department of Pathology, Kangbuk Samsung Hospital, Sungkyunkwan University School of Medicine, Seoul 03181, Korea; swan.chae@samsung.com; 4 Seoul Jun Research Center, Seoul Jun Rehabilitation Clinic, Seoul 06737, Korea; cathedral.sjk@gmail.com; 5 Department of Physical and Rehabilitation Medicine, Kangbuk Samsung Hospital, Sungkyunkwan University School of Medicine, Seoul 03181, Korea

**Keywords:** tendinopathy, tendon-derived stem cells, diabetes, macrophage migration inhibitory factor

## Abstract

The purpose of our study was to evaluate the role of macrophage migration inhibitory factor (MIF) in the differentiation of tendon-derived stem cells (TdSCs) under hyperglycemic conditions. In the in vivo experiment, rats were classified into diabetic (DM) and non-DM groups depending on the intraperitoneal streptozotocin (STZ) or saline injection. Twelve-week after STZ injection, the supraspinatus tendon was harvested and prepared for histological evaluation and real-time reverse transcription polymerase chain reaction for osteochondrogenic (aggrecan, BMP-2, and Sox9) and tenogenic (Egr1, Mkx, scleraxis, type 1 collagen, and Tnmd) markers. For the in vitro experiment, TdSCs were isolated from healthy rat Achilles tendons. Cultured TdSCs were treated with methylglyoxal and recombinant MIF or MIF gene knockdown to determine the effect of hyperglycemic conditions and MIF on the differentiation function of TdSCs. These conditions were classified into four groups: hyperglycemic-control group, hyperglycemic-recombinant-MIF group, hyperglycemic-knockdown-MIF group, and normal-control group. The mRNA expression of osteochondrogenic and tenogenic markers was compared among the groups. In the in vivo experiment, the mRNA expression of all osteochondrogenic and tenogenic differentiation markers in the DM group was significantly higher and lower than that in the non-DM group, respectively. Similarly, in the in vitro experiments, the expression of all osteochondrogenic and tenogenic differentiation markers was significantly upregulated and downregulated, respectively, in the hyperglycemic-control group compared to that in the normal-control group. The hyperglycemic-knockdown-MIF group demonstrated significantly decreased expression of all osteochondrogenic differentiation markers and increased expression of only some tenogenic differentiation markers compared with the hyperglycemic-control group. In contrast, the hyperglycemic-recombinant-MIF group showed significantly increased expression of all osteochondrogenic differentiation markers, but no significant difference in any tenogenic marker level, compared to the hyperglycemic-control group. These results suggest that tendon homeostasis could be affected by hyperglycemic conditions, and MIF appears to alter the differentiation of TdSCs via enhancement of the osteochondrogenic differentiation in hyperglycemic conditions. These are preliminary findings, and must be confirmed in a further study.

## 1. Introduction

Tendinopathy is a common musculoskeletal pathology that causes pain and dysfunction and commonly occurs around the shoulder, elbow, and ankle joints [1]. In molecular terms, tendinopathy refers to a pathological condition in which disrupted tendon healing (degeneration) overwhelms the normal healing process (repair) in the tendon after acute or repetitive cumulative injury [2]. The normal tendon healing process involves three sequential and overlapping phases: inflammation, proliferation, and remodeling [3,4]. Overwhelming of the healing process as with disrupted tendons can progress to chronic mucoid and/or lipoid degeneration of the tendon with a variable amount of fibrocartilaginous metaplasia and calcium hydroxyapatite deposits [5,6,7]. There is a clear clinical association between diabetes and various tendinopathies such as rotator cuff tendinopathy, lateral epicondylitis, and Achilles tendinopathy [8,9,10,11]. The degenerative features of the Achilles tendon on ultrasonography in type 2 diabetes were approximately 50%, while those in the control group were approximately 18.8% [12]. Tendinopathy in diabetes is a complex, multifactorial, pathological process involving altered macrophage function, angiogenic signaling, and neurotrophic signaling under both a chronic, low-grade inflammation and a high-glucose environment [13,14,15,16].

Diabetes inhibits tendon homeostasis and adversely affects the healing process after acute-phase injury [17]. For instance, the re-rupture rate after rotator cuff repair in diabetes patients is up to two times higher than that in patients without diabetes [18,19]. The effect of diabetes on tendon function is well known, although the underlying mechanism remains unclear. The proposed pathogenesis of tendinopathy in diabetes has been described in terms of advanced glycation end products (AGEs), which are oxidative derivatives resulting from diabetic hyperglycemia. These could be deposited in collagen-rich tissue such as tendons and serve as connectors between free amino groups of neighboring proteins to form intermolecular crosslinks, thereby causing increased tissue stiffness and a subsequent predisposition to tendinopathy [20]. A recent study demonstrated that a high glucose environment in local tendon cells may dysregulate the resolution of inflammation [21]. Abnormal, excessive, or insufficient inflammation has serious detrimental effects on the tendon healing process [13].

Tendon-derived stem cells (TdSCs) constitute a minor cell population in the tendon and can be isolated from tendon tissues. TdSCs exhibit the universal stem cell characteristics of self-renewal and multi-lineage differentiation capacities including differentiation into tendon-like tissues, even after extended in vitro and in vivo expansion [22]. TdSCs are crucial for tendon repair and homeostasis [23]. TdSCs isolated from a failed tendon healing model exhibit increased osteochondrogenic differentiation potential compared to the tenogenic process [24].

Macrophage migration inhibitory factor (MIF) is a potent and pleiotropic cytokine secreted by activated T cells and macrophages that plays a critical role in inflammatory and autoimmune diseases [25]. In addition, MIF has been identified as an important regulator of the pathogenesis of diabetes. Recent studies have reinforced the evidence of an association between MIF and diabetes-related clinical complications, such as neuropathy, nephropathy, and retinopathy [26,27,28,29]. However, the effect of MIF on tendon homeostasis in diabetic conditions has not been elucidated yet.

Therefore, in this study, we aimed to confirm the harmful effects of diabetes on tendon homeostasis and to clarify the effect of MIF on the differentiation potential and tendon homeostasis of TdSCs under hyperglycemic conditions. We hypothesized that the expression of osteochondrogenic and tenogenic genes would be upregulated and downregulated, respectively, in hyperglycemic conditions compared to non-hyperglycemic (normal) conditions. We further predicted that MIF would enhance the osteochondrogenic differentiation of TdSCs under hyperglycemic conditions, along with a concomitant inhibition of tenogenic differentiation.

## 2. Results

### 2.1. Confirmation of Streptozotocin(STZ)-Induced Diabetes(DM) Rat Model

We serially measured body weight and blood glucose levels for 12 weeks (Supplementary Materials). Blood glucose levels in the non-DM group were maintained under 270 mg/dL until the 12th week. The DM group maintained hyperglycemic states until the 12th week (DM group: 476.5 ± 31.5, sham (non-DM) group: 109.8 ± 7.1, *p* < 0.001).

### 2.2. In Vivo Animal Experiments: Comparative Histological Analysis of DM and Non-DM Groups

**Figure 1:**
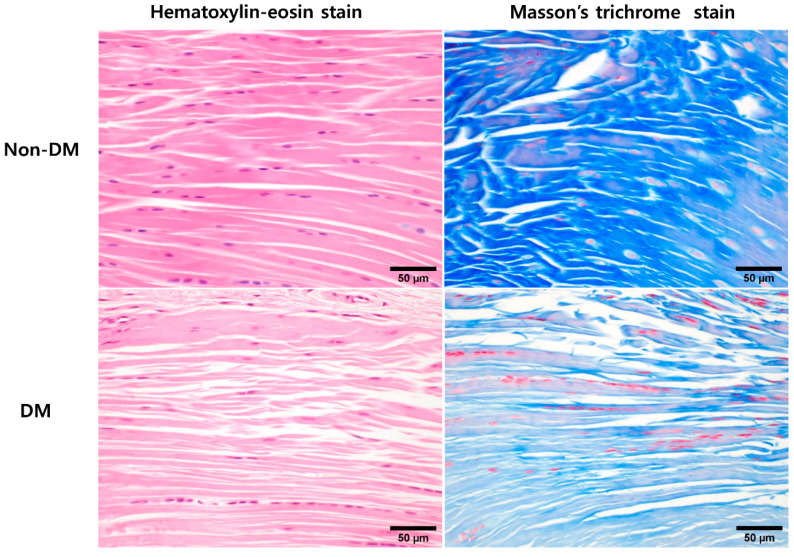
Comparison of histological analysis of supraspinatus tendon harvested from the diabetic (DM) and non-DM groups 12-week after STZ treatment. DM group showed slightly decreased cellularity of tenocytes, thinner fiber thickness with larger interfibrillar spaces and poorly organized collagen fibers compared to non-DM group. Masson’s trichrome staining showed more densely blue-stained collagen bundles in non-DM group compared to that of DM group.

Twelve-week after STZ treatment, histologically, supraspinatus tendons tended to have spindle-shaped nuclei of tenocytes and slightly decreased cell density in the DM group. In contrast, the nuclei of non-DM tenocytes were oval to spindle shaped with a uniform cell distribution. The DM group also showed thinner fibers with larger interfibrillar spaces and poorly organized collagen fibers compared with the non-DM group (Figure 1).

### 2.3. In Vivo Animal Experiments: Effect of Diabetic Condition on Supraspinatus Tendon

**Figure 2:**
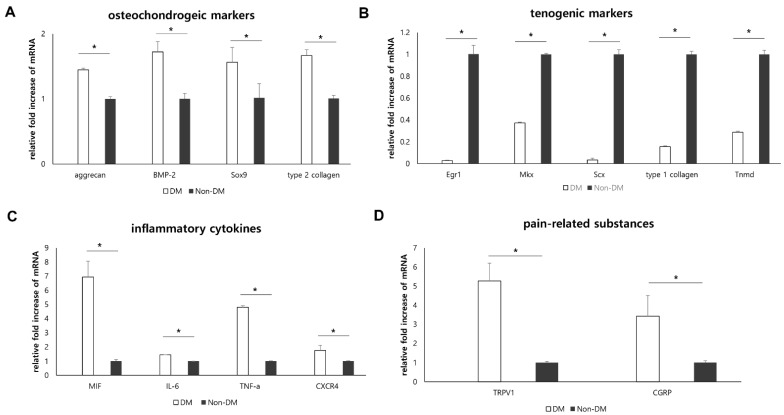
Effect of diabetes on the mRNA expression levels of osteochondrogenic markers, tenogenic makers, inflammatory cytokines, and pain-related substances in supraspinatus tendons in vivo. Changes in mRNA expression levels of (**A**) osteochondrogenic differentiation markers (aggrecan, bone morphogenic protein (BMP)-2, Sox9, and type 2 collagen), (**B**) tenogenic differentiation markers (Egr1, Mkx, Scx, type 1 collagen, and tenomodulin (Tnmd)), (**C**) inflammatory cytokines (macrophage migration inhibitory factor (MIF), interleukin (IL)-6, tumor necrosis factor (TNF)-α, and CXCR4, and (**D**) pain-related substances (calcitonin gene-related peptide (CGRP) and transient receptor potential vanilloid 1 (TRPV1)) in diabetic (DM) and non-DM groups. The mRNA expression levels of the target genes were normalized to that of the GAPDH gene. *: statistical significance between the diabetic (DM) and non-DM groups.

Twelve-week after the STZ treatment, we quantified the mRNA expression levels of osteochondrogenic differentiation markers, tenogenic differentiation markers, inflammatory cytokines, and pain-related substances. The mRNA expression of osteochondrogenic differentiation markers (aggrecan, bone morphogenic protein (BMP)-2, type 2 collagen, and Sox9) was significantly lower in the non-DM group than in the DM group (*p =* 0.021 for aggrecan, *p =* 0.020 for BMP-2, *p =* 0.021 for type 2 collagen, and *p =* 0.021 for Sox9; Figure 2A). The mRNA expression levels of tenogenic differentiation markers (early growth response 1 (Egr1), mohawk (Mkx), scleraxis (Scx), type 1 collagen, and tenomodulin (Tnmd)) were significantly downregulated in the DM group compared to those in the non-DM group (*p =* 0.021 for Erg1, *p =* 0.021 for Mkx, *p =* 0.021 for Scx, *p =* 0.020 for type 1 collagen, and *p =* 0.021 for Tnmd; Figure 2B). The expression of inflammatory cytokines (MIF, tumor necrosis factor (TNF)-α, and interleukin (IL)-6) and functional receptors for MIF (C-X-C chemokine receptor type 4 (CXCR4)) was significantly upregulated in the DM group compared to that in the non-DM group (*p =* 0.021 for MIF, *p =* 0.021 for TNF-α, *p =* 0.013 for IL-6, and *p =* 0.021 for CXCR4; Figure 2C). The expression of mRNA coding for pain-related substances (such as calcitonin gene-related peptide (CGRP) and transient receptor potential vanilloid-1 (TRPV1)) was significantly higher in the DM group than in the non-DM group (*p =* 0.021 for CGRP and *p =* 0.021 for TRPV1; Figure 2D).

### 2.4. In Vitro Experiments: Effect of MIF on TdSCs under Non-Hyperglycemic Condition

**Figure 3:**
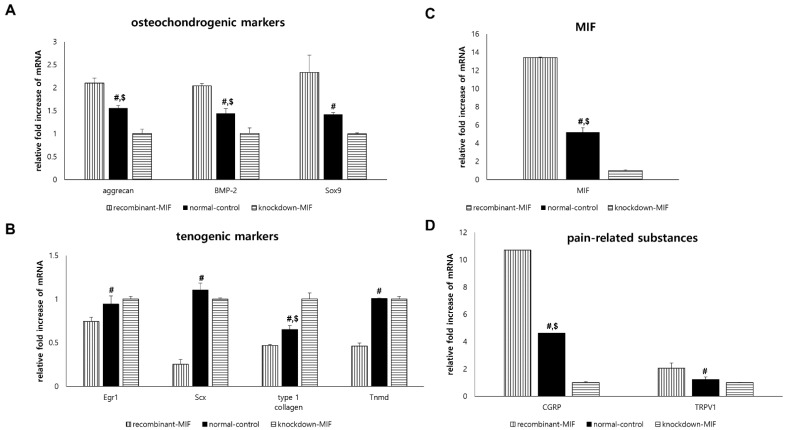
Effect of macrophage migration inhibitory factor (MIF) on TdSCs under non-diabetic condition in vitro. Comparative analysis of mRNA expression levels of (**A**) osteochondrogenic differentiation markers (aggrecan, bone morphogenic protein (BMP)-2, and Sox9), (**B**) tenogenic differentiation markers (Egr1, Scx, type 1 collagen, and Tnmd), (**C**) MIF and (**D**) pain-related substances (CGRP and TRPV1) in TdSCs. The mRNA expression levels of the target genes were normalized to that of the GAPDH gene. ^#^: statistical significance between the normal-control and recombinant-MIF groups; ^$^: statistical significance between the normal-control and knockdown-MIF groups.

Compared with the normal-control group, the expression of osteochondrogenic differentiation markers (aggrecan, BMP-2, and Sox9) was significantly increased in the recombinant-MIF group (*p* < 0.001 for aggrecan and BMP-2, *p =* 0.001 for Sox9) and tended to be decreased in the knockdown-MIF group (*p* < 0.001 for aggrecan and BMP-2, *p =* 0.068 for Sox9; Figure 3A).

Compared with the normal-control group, the expression of tenogenic differentiation markers (Egr1, Scx, type 1 collagen, and Tnmd) was significantly decreased in recombinant-MIF (*p =* 0.005 for Egr1, *p* < 0.001 for Scx, *p =* 0.001 for type 1 collagen, and *p* < 0.001 for Tnmd). The knockdown-MIF group showed mRNA overexpression for only a subset of the tenogenic markers (*p =* 0.719 for Egr1, *p =* 0.085 for Scx, *p* < 0.001 for type 1 collagen, and *p =* 1.000 for Tnmd; Figure 3B) compared to the normal-control group.

Compared with the normal-control group, the mRNA expression level of MIF was significantly increased in the recombinant-MIF group (*p* < 0.001) and was decreased in the knockdown-MIF group (*p* < 0.001; Figure 3C).

Compared with those in the normal-control group, the mRNA expression levels of pain-related markers (CGRP and TRPV1) were significantly upregulated in the recombinant-MIF group (*p* < 0.001 for CGRP, *p =* 0.003 for TRPV1) and only CGRP was significantly downregulated in the knockdown-MIF group (*p =* 0.003 for CGRP, *p =* 0.736 for TRPV1; Figure 3D).

### 2.5. In Vitro Experiments: Effect of Hyperglycemic Condition and MIF on TdSCs

**Figure 4:**
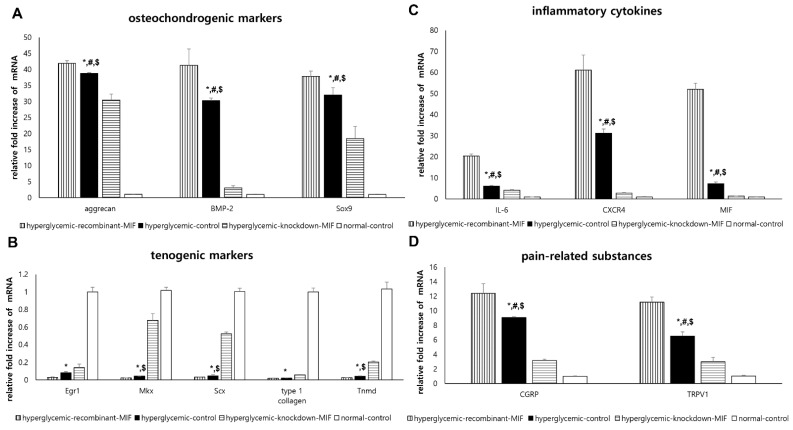
Effect of diabetic status and macrophage migration inhibitory factor (MIF) on TdSCs in vitro. Comparative analysis of mRNA expression levels of (**A**) osteochondrogenic differentiation markers (aggrecan, bone morphogenic protein (BMP)-2, and Sox9), (**B**) tenogenic differentiation markers (Egr1, Mkx, Scx, type 1 collagen, and Tnmd), (**C**) inflammatory cytokines (IL-6, CXCR4, and MIF), and (**D**) pain-related substances (CGRP and TRPV1) in TdSCs. The mRNA expression levels of the target genes were normalized to that of the GAPDH gene. *: statistical significance between the hyperglycemic-control and normal-control groups; ^#^: statistical significance between the hyperglycemic-control and hyperglycemic-recombinant-MIF groups; ^$^: statistical significance between the hyperglycemic-control and hyperglycemic-knockdown-MIF groups.

There were significant differences among the four groups (hyperglycemic-control, hyperglycemic-recombinant-MIF, hyperglycemic-knockdown-MIF, and normal-control) with respect to the expression of all osteochondrogenic differentiation markers (*p* < 0.001 for all). The mRNA expression levels of these markers (aggrecan, BMP-2, and Sox9) were significantly higher in the hyperglycemic-control group than in the normal-control group (*p* < 0.001 for all) (Figure 4A). Compared with that in the hyperglycemic-control group, the expression of all osteochondrogenic differentiation markers was significantly increased in the hyperglycemic-recombinant-MIF group (*p =* 0.006 for aggrecan, *p* < 0.001 for BMP-2, and *p =* 0.031 for Sox9) and significantly decreased in the hyperglycemic-knockdown-MIF group (*p* < 0.001 for all; Figure 4A).

Similarly, there were significant differences among the four groups in terms of all the tenogenic markers studied (*p* < 0.001 for all tenogenic markers). The mRNA expression of the tenogenic differentiation markers (Egr1, Mkx, Scx, type 1 collagen, and Tnmd) was significantly lower in the hyperglycemic-control group than in the normal-control group (*p* < 0.001 for all; Figure 4B). None of the tenogenic markers showed significant differences in the mRNA expression levels between the hyperglycemic-control group and the hyperglycemic-recombinant-MIF group (*p =* 0.263 for Egr1, *p* = 0.901 for Mkx, *p =* 0.863 for Scx, *p =* 1.000 for type 1 collagen, and *p =* 0.950 for Tnmd). Additionally, only some tenogenic markers in the hyperglycemic-knockdown-MIF group showed mRNA overexpression (*p* < 0.001 for Mkx; *p* < 0.001 for Scx, *p =* 0.001 for Tnmd, *p* = 0.207 for Egr1, and *p =* 0.202 for type 1 collagen; Figure 4B) compared with the hyperglycemic-control group.

There were significant differences among the four groups for all inflammatory cytokines (*p* < 0.001 for all). The mRNA expression of IL-6, CXCR4, and MIF was significantly higher in the hyperglycemic-control group than in the normal-control group (*p* < 0.001 for all; Figure 4C). Compared with those in the hyperglycemic-control group, the mRNA expression levels of IL-6, CXCR4, and MIF were significantly upregulated in the hyperglycemic-recombinant-MIF group (*p* < 0.001 for all) and significantly downregulated in the hyperglycemic-knockdown-MIF group (*p =* 0.002 for IL-6, *p* < 0.001 for CXCR4, and *p* < 0.001 for MIF; Figure 4C).

There were significant differences among the four groups for all pain-related substances (*p* < 0.001 for all). The mRNA expression of CGRP and TRPV1 was significantly higher in the hyperglycemic-control group than in the normal-control group (*p* < 0.001 for all; Figure 4D). Compared with those in the hyperglycemic-control group, the mRNA expression levels of CGRP and TRPV1 were significantly upregulated in the hyperglycemic-recombinant-MIF group (*p* < 0.001 for all) and significantly downregulated in the hyperglycemic-knockdown-MIF group (*p =* 0.002 for all; Figure 4D).

## 3. Discussion

The mechanisms underlying the pathogenesis of tendinopathy in diabetes remain unclear [30]. The novel finding in this study is that MIF can alter the tendon differentiation function of TdSCs in hyperglycemic conditions. Under hyperglycemic conditions, expression of osteochondrogenic differentiation markers in TdSCs was upregulated following treatment with recombinant MIF and downregulated upon knockdown of the MIF gene. In contrast, treatment with recombinant MIF did not affect the expression of tenogenic differentiation markers, and knockdown of the MIF gene upregulated the mRNA expression level of only some of the tenogenic differentiation markers. To the best of our knowledge, this is the first study on the effects of hyperglycemic conditions and MIF on the differentiation function of TdSCs.

Aberrant differentiation of TdSCs toward osteochondrogenesis has been linked to the pathogenesis of chronic tendinopathy [24]. Previous studies also reported that TdSCs in diabetic conditions tended to erroneously differentiate toward osteochondrogenesis rather than tenogenesis, which explains the pathogenesis of tendon disorders in diabetes [11,23,24]. Similarly, the present in vivo study demonstrated that the expression of osteochondrogenic markers, including aggrecan, BMP-2, type 2 collagen, and Sox9, were upregulated in the supraspinatus tendon of the DM group, whereas that of tenogenic markers (Egr1, Mkx, Scx, type 1 collagen, and Tnmd) decreased, compared to those in the non-DM group. These results validated that diabetic conditions could alter tendon homeostasis and bias TdSCs toward osteochondrogenesis (or non-tenogenic aberrant differentiation).

We further investigated factors that could play a major role in the erroneous differentiation of TdSCs under hyperglycemic conditions. MIF is an important mediator in the development of diabetes and various associated complications. The MIF/CD74 signaling pathway promotes macrophage-mediated inflammation in type 1 diabetes, and MIF controls the recruitment of inflammatory cells via chemokine receptors CXCR2 and CXCR4 [31,32]. We previously reported that MIF can aggravate diabetic neuropathy by suppressing glyoxalase I and intraepidermal nerve fibers on the footpad skin [28]. Another study reported the involvement of the MIF/CD74 signaling axis in proliferative diabetic retinopathy, suggesting that MIF expression is related to retinal neovascularization [26]. In fact, neovascularization is a prominent histological characteristic of tendinopathy [1,5,17]. Recent research has also revealed a positive correlation between MIF concentration in the lumbar ligamentum flavum and its thickness in patients with type 2 diabetes and spinal stenosis [33]. However, the effect of MIF on tendon homeostasis in diabetic conditions has not been established yet.

To identify whether MIF affects tendon homeostasis in diabetes, we designed an in vitro experimental model using TdSCs harvested from the Achilles tendon of healthy SD rats and assessed changes in differentiation potential with MIF exposure in experimental hyperglycemic conditions. Our in vitro experiments under hyperglycemic conditions showed that the expression of osteochondrogenic and tenogenic differentiation markers was significantly increased and decreased in hyperglycemic condition (hyperglycemic-control group) compared to that in non-hyperglycemic normal condition (normal-control group). This finding suggested that the hyperglycemic condition itself is a major determinant of TdSCs differentiation that strongly enhanced osteochondrogenic differential potential and suppressed the tenogenic differentiation potential. There would be many unknown underlying mechanisms involved in this finding. Our in vivo study showed diabetic condition affected the tendon homeostasis (Figure 1) and MIF was significantly up-regulated in tendons of diabetic rat (Figure 2C). However, the effect of MIF on tendon homeostasis in diabetic conditions has not been established yet. Thus, we attempted to investigate the modifiable factors under hyperglycemic condition, such as MIF, to modulate the erroneous differentiation of TdSCs through an in vitro experiment. Our in vitro study showed that under hyperglycemic condition, knockdown of the MIF gene was more likely to affect the osteochondrogenic differentiation where all osteochondrogenic markers were down-regulated (Figure 4A) compared to tenogenic differentiation where only some tenogenic markers were up-regulated (Figure 4B). In addition, under hyperglycemic condition, the expression of all osteochondrogenic differentiation markers significantly changed depending on the administration of recombinant MIF (Figure 4A), while none of the tenogenic markers was affected by the administration of recombinant MIF (Figure 4B). These findings suggest that MIF is more likely to augment osteochondrogenic differentiation rather than inhibit tenogenic differentiation under hyperglycemic conditions. This is probably due to the kind of ‘ceiling effect’ that the hyperglycemic condition itself sufficiently inhibited tenogenic differentiation so that there was no room for additional effect of recombinant MIF. Thus, under hyperglycemic condition, knockdown of the MIF gene can be a therapeutic target for the treatment or prevention of diabetic tendinopathy in the future.

TdSCs exhibit a spontaneous tenogenic differentiation function, which plays an important role in tendon homeostasis and repair. Therefore, the results of the in vitro experiments may account for the findings of the in vivo study reported here, where expression of osteochondrogenic markers (aggrecan, BMP-2, type 2 collagen, and Sox9) increased, while that of tenogenic markers (Egr1, Mkx, Scx, Tnmd, and type 1 collagen) decreased in diabetic tendons compared to those in normal tendons. Additionally, these results are in line with previous reports that microscopic chondroid metaplasia is frequently seen in diabetic tendinopathy, and calcium deposits are more common in the Achilles, supraspinatus, and common extensor tendon of the wrist in diabetes patients [30,34,35]. Our in vivo study also demonstrated that the histological findings of the DM group showed slightly decreased cellularity of tenocytes, thinner fiber thickness with larger interfibrillar spaces, and poorly organized collagen fibers when compared to the non-DM group.

Based on these findings, hyperglycemic conditions appear to play a major role in the erroneous differentiation of TdSCs, and MIF likely amplifies this effect via enhancement of osteochondrogenic differentiation of TdSCs in hyperglycemic conditions. Although the in vitro evidence of the effect of MIF on the differentiation of TdSCs, it is still unclear whether MIF can alter the tissue microenvironment, for instance, the composition of the extracellular matrix, migration of tendon fibroblasts, and tenocyte proliferation in vivo, as expected. The erroneous ratio between tenogenic and osteochondrogenic differentiation of TdSCs in an in vitro experiment might provide clues to the pathogenesis of tendinopathy in DM. Future studies are necessary to confirm the effect of MIF itself on in vivo diabetic tendons using MIF-null mice.

The expression levels of markers for inflammation and pain transmission, including IL-6, CXCR4, CGRP, and TRPV1, showed significant changes depending on the presence of MIF in the hyperglycemic conditions simulated in this study. MIF is known to induce the expression of key proinflammatory genes, including IL-1, TNF, IL-6, IL-8, COX-2, and MMPs, and has been implicated in the development of many acute inflammatory and autoimmune diseases as well as chronic inflammatory metabolic disorders [31,36,37]. Additionally, MIF may have multiple unknown functions in various diseases. Previous research has suggested the role of MIF in the development of pain hypersensitivity by demonstrating that MIF null mice did not develop hypersensitivity after nerve injury, and pharmacological inhibition of MIF in wild mice suppressed hypersensitivity [38]. Our previous study also showed that the expression of MIF was increased in the footpad skin lesions of diabetic rats. MIF, therefore, seems to be an important upstream mediator of factors contributing to pain [28]. However, there have been few studies of pain-related factors in diabetic tendinopathy, and the function of MIF in the pain process is yet to be elucidated. Our results revealed that the expression of pain-related substances in TdSCs was dependent on the treatment of recombinant MIF or knockdown of MIF, implicating MIF as a regulator of pain in diabetic tendinopathy.

The main limitation of this study is that the molecular basis of the observed MIF-mediated enhancement of the erroneous differentiation potential in TdSCs has not been fully identified. Therefore, further studies on the various steps of MIF-related signaling are needed. We did not survey the effects of knockdown MIF on the TdSCs isolated from diabetic rats. If we could show that knockdown MIF has a beneficial effect on osteochondrogenic and tenogenic differentiation of the TdSCs isolated from diabetic rats, that would be a strong evidence for arguing that MIF has a role in the pathogenesis of the tendon defect in those with diabetes. In addition, we did not perform an in vivo study using MIF null mice to identify the effect of MIF on the differentiation potential of TdSCs under hyperglycemic conditions. In our in vivo study, there was a modest decrease in the expression of the differentiation markers of diabetic rats. The differences between the two groups in differentiation markers would have become clearer if the duration of hyperglycemic exposure had been longer. In the in vivo study, we used the STZ-induced type 1 diabetes rat model, although tendinopathies are usually associated with type 2 diabetes. The type 2 diabetes rat model might be more appropriate than the type 1 diabetes model to better understand the effects of type 2 diabetes on tendon homeostasis. However, a previous study reported that the STZ-induced type 1 diabetes rat model also demonstrated the disruption of tendon healing [14]. We analyzed only the mRNA levels of osteochondrogenic, tenogenic, and inflammatory markers. The results of PCR data alone are insufficient to conclude the effect of MIF on TdSC differentiation. Further studies are needed to investigate the protein expression of osteochondrogenic, tenogenic, and inflammatory markers as well as extracellular matrix protein expression using Western blotting or immunohistochemistry staining. Our study did not investigate the expression of metalloproteinases and tissue inhibitors of metalloproteinases that regulate collagenous and non-collagenous matrix production. We also did not survey the expression of adipogenic markers in TdSCs even though lipid deposits have been observed in degenerated tendons.

## 4. Materials and Methods

### 4.1. Experimental Animals

Twenty-four Sprague–Dawley (SD) rats (male, 5-week-old, 160–200 g) were obtained from Orient Bio. Inc. (Gapyeong, South Korea). Rats were housed in pairs, allowed ad libitum access to standard lab chow and water, and maintained on a 12-h light–dark cycle at a constant temperature (22 ± 1 °C).

### 4.2. In Vivo Animal Experiments: Type 1 Diabetic Rat Model

After 2 weeks of acclimation, 12 rats (250–300 g, 7-week-old) were randomly divided into diabetes (DM) and non-DM groups. Diabetes was induced by a single intraperitoneal injection of streptozotocin (STZ; 70 mg/kg, Merck Chemicals, Feldbergstraβe, Darmstadt, Germany) freshly dissolved in saline. The non-DM group was administered a single intraperitoneal injection of phosphate-buffered saline. Following the injections, all rats were kept in cages in pairs with unrestricted activity and food and water ad libitum in a specific pathogen-free environment, with each experimental group placed in a separate cage. Blood glucose levels and body weights were checked weekly throughout the 12-week period after STZ injection. Blood glucose levels were evaluated in blood samples from the tail veins using a glucometer (ACCU-CHEK, Roche, Nutley, NJ, USA). Animals with blood glucose levels >270 mg/dL were considered diabetic. None of the rats died or showed any evidence of illness during the study period. This study was approved by the Ethics Committee for Animal Experiments at Kangbuk Samsung Hospital (approval number: 16-082-D3-N). The animal experiments were conducted in accordance with the ethical guidelines of the International Council for Laboratory Animal Science.

### 4.3. In Vivo Animal Experiments: Tissue Sampling

Twelve weeks after STZ injection, all rats were sacrificed for tissue sampling. The rats were anesthetized with isoflurane and perfused transcardially with saline under aseptic conditions. The supraspinatus tendon was harvested and prepared for histological analysis and real-time reverse transcription polymerase chain reaction (RT-PCR). After the appropriate skin incision, the deltoid was split, and the acromioclavicular joint was divided to reveal the rotator cuff. The supraspinatus tendon was identified and cut off at its point of insertion into the greater tuberosity. One side of the supraspinatus tendon attached to its bony insertion on the humerus was immediately fixed with 10% neutral-buffered formalin (BBC Biochemical, Mount Vernon, WA, USA) for histological analysis. The other side of the supraspinatus tendon was snap-frozen in liquid nitrogen and stored at −80 °C for RT-PCR.

### 4.4. In Vivo Animal Experiments: Supraspinatus Tendon Histology

After fixing in 10% neutral-buffered formalin, bone–tendon–muscle units were decalcified in a decalcifying solution (TBD-1 decalcifier, Thermo Scientific, Kalamazoo, MI, USA). The decalcification process was terminated when the bone could be penetrated using a needle without any force. Subsequently, specimens were embedded in paraffin, and 4-µm sections were cut and placed on silane-coated slides (Muto Pure Chemicals, Tokyo, Japan). The deparaffinized sections were stained with hematoxylin-eosin and Masson’s trichrome stain. These sections were then examined by light microscopy to assess the morphology of tenocytes, cellularity, and structure or arrangement of collagen fibers.

### 4.5. In Vitro Experiments: TdSCs Culture

TdSCs were isolated from the Achilles tendon of healthy SD rats (male, 5-week-old), as previously described by Kim et al. [39] wherein we successfully isolated and identified TdSCs, and determined the stem cell properties of TdSCs, the multipotent capacity using fluorescence-activated cell sorting (FACS) analysis, immunofluorescent staining and culture in specific differentiation media, etc. Briefly, under aseptic conditions, the rats were anesthetized with isoflurane and perfused transcardially with saline to prevent blood contamination. The bilateral Achilles tendons were separated, and the tendon sheaths and paratendons were carefully removed. The tendons were washed using a stepwise dilution of culture media and diluted with five-fold aliquots of antibiotics. Next, the Achilles tendon tissues were minced into approximately 1 × 1 mm pieces and digested in saline supplemented with 3 mg collagenase type I (C0130, Merck Chemicals) and 4 mg dispase (D4693, Merck Chemicals) per 100 mg tissue. The samples were incubated at 37 °C for 2 h and then centrifuged at 2000× *g* rpm for 15 min. The cell pellets were rinsed and resuspended in Dulbecco’s modified Eagle’s medium (DMEM; Thermo Fisher Scientific Inc., Waltham, MA, USA) with 20% fetal bovine serum (FBS; Thermo Fisher Scientific Inc.) and 1% penicillin/streptomycin (Thermo Fisher Scientific Inc.). To isolate TdSCs, 100 cells were seeded in a 100-mm dish and cultured in DMEM containing 20% FBS and 1% penicillin/streptomycin. The TdSCs were identified based on colony formation. After 8–10 days, individual cell colonies were marked on the surface of the 100-mm dish using a light microscope. TdSCs were collected by localized trypsin treatment in each area demarcating a colony using a micropipette, and this step was defined as passage 0 of TdSCs. The isolated TdSCs were sub-cultured and passaged when they reached 80% confluence. Passage three of the TdSCs was used for our experiments.

### 4.6. In Vitro TdSCs Experiments under Non-Hyperglycemic Normal Conditions: Recombinant MIF Treatment vs. Small Interference RNA Transfection

TdSCs were cultured in DMEM supplemented with 20% FBS and 1% penicillin/streptomycin in a humidified atmosphere of 5% CO_2_ at 37 °C. These cells were sub-cultured and seeded onto six-well plates (Nunc, Naperville, IL, USA) at a density of 200,000 cells per plate. The experiments were performed when the cells reached approximately 80% confluence. To determine the effect of MIF on TdSCs, recombinant MIF (10 ng/mL, ProSpec, East Brunswick, NJ, USA) was added to TdSCs (recombinant-MIF group), and MIF gene knockdown was performed with MIF small interfering RNA (OriGene Technologies, Rockville, MD, USA) using the Lipofectamine RNAiMAX reagent (Thermo Fisher Scientific Inc.) according to the manufacturer’s protocol (knockdown-MIF group). TdSCs cultured with only the culture medium were used as normal conditions (normal-control group). Therefore, there were three experimental groups: the recombinant-MIF, knockdown-MIF, and normal-control.

### 4.7. In Vitro TdSCs Experiments under Hyperglycemic Condition: Recombinant MIF Treatment vs. Small Interference RNA Transfection for TdSCs

TdSCs were cultured in DMEM supplemented with 20% FBS and 1% penicillin/streptomycin in a humidified atmosphere of 5% CO_2_ at 37 °C. These cells were sub-cultured and seeded onto six-well plates (Nunc, Naperville, IL, USA) at a density of 200,000 cells per plate. The experiments were performed when the cells reached approximately 80% confluence. To mimic hyperglycemic conditions in vitro, each culture plate was treated with 400 μM methylglyoxal (Merck Chemicals), followed by incubation for 12 h (hyperglycemic-control group). To determine the effect of MIF on TdSCs maintained in hyperglycemic conditions, recombinant MIF (10 ng/mL, ProSpec, East Brunswick, NJ, USA) was added to TdSCs following treatment with methylglyoxal (hyperglycemic-recombinant-MIF group), and MIF gene knockdown was performed with MIF small interfering RNA (OriGene Technologies, Rockville, MD, USA) using the Lipofectamine RNAiMAX reagent (Thermo Fisher Scientific Inc.) according to the manufacturer’s protocol following treatment with methylglyoxal (hyperglycemic-knockdown-MIF group). TdSCs cultured with only culture medium were used as the -normal conditions (normal-control group). Therefore, there were four experimental groups in the in vitro study: hyperglycemic-control, hyperglycemic-recombinant-MIF, hyperglycemic-knockdown-MIF, and normal-control. The concentration of methylglyoxal and MIF was determined based on the protocol described in our previous study [28].

### 4.8. In Vivo Animal Experiments & In Vitro TdSCs Experiments: RT-PCR

Total RNA was extracted from supraspinatus tendon tissue and TdSCs by lysis in TRIzol reagent (Thermo Fisher Scientific Inc.) according to the manufacturer’s protocol. An aliquot of 10 µg total RNA was prepared from each sample and reverse-transcribed into cDNA using M-MLV reverse transcriptase (Promega, Madison, WI, USA) in 20 µL of reaction buffer at 42 °C for 2 h. The cDNA was amplified with 2x SYBR Green Master Mix kit (Roche Diagnostics, Indianapolis, IN, USA) using a Light Cycler 480 real-time PCR detection system (Roche Diagnostics) with the following thermal cycling program: 40 cycles of step 1 at 94 °C for 30 s; step 2 at 60 °C for 30 s; and step 3 at 72 °C for 40 s. Relative gene expression was calculated using the 2^−ΔΔCT^ method (n = 4 per group). Expression of the target gene was normalized to that of GAPDH. The target primer sequences are listed in Table 1.

### 4.9. Statistical Analysis

All data are expressed as mean ± standard deviation. IBM Statistical Package for Social Sciences (SPSS) version 21 software (Armonk, New York, NY, USA) was used for data analysis. For in vivo study, body weight and blood glucose level at each point and mRNA expression levels for each parameter were compared between the DM and non-DM groups using the Mann–Whitney U test. For in vitro study, differences among groups for each parameter were determined by repeated measures analysis of variance, and post-hoc comparisons between two groups were performed using the Bonferroni method. Differences were considered statistically significant at *p* < 0.05.

## 5. Conclusions

Tendon homeostasis can be affected by diabetes due to decreased tenogenic differentiation and increased osteochondrogenic differentiation of TdSCs. Under hyperglycemic condition, MIF appears to enhance the osteochondrogenic differentiation of TdSCs, rather than suppressing tenogenic differentiation. These are preliminary findings, and must be confirmed in a further study.

## Figures and Tables

**Table 1:** Primer sequences for the rat genes used in the study.

	Forward	Reverse
Aggrecan	5′-AAGTGCTATGCTGGCTGGTT-3′	5′-GGTCTGGTTGGGGTAGAGGT-3′
BMP-2	5′-TAGTGACTTTTGGCCACGACG-3′	5′-GCTTCCGCTGTTTGTGTTTG-3′
Sox9	5′-CTGCGACCTCAGAAGGAAAG-3′	5′-CGCTGGTATTCAGGGAGGTA-3′
Erg1	5′-TTATCCCAGCCAAACTACCC-3′	5′-CAGAGGAAGACGATGAAGCA-3′
type 2 collagen	5′-CTCAAGTCGCTGAACAACCA-3′	5′-GTCTCCGCTCTTCCACTCTG-3′
Mkx	5′-CTCCGGTTTCGATTGAGAAG-3′	5′-AGTTGTTCACGGCTCTTCGT-3′
Scx	5′-AACACGGCCTTCACTGCGCTG-3′	5′-CAGTAGCACGTTGCCCAGGTG-3′
Tnmd	5′-GTGGTCCCACAAGTGAAGGT-3′	5′-GTCTTCCTCGCTTGCTTGTC-3′
type 1 collagen	5′-TGGAGACAGGTCAGACCTG-3′	5′-TATTCGATGACTGTCTTGCC-3′
CGRP	5′-CTGTCGAGTTTCCACAGGTTCC-3′	5′-TTGCGCAGAGGTACGGTTCC-3′
TRPV1	5′- CCACAGCGGTGGTGACGC -3′	5′- GGAGCTGTCAGGTGGCCG -3′
CXCR4	5′-ATCATCTCCAAGCTGTCACACTCC-3′	5′-GTGATGGAGATCCACTTGTGCAC-3′
MIF	5′-CCCAGAACCGCAACTACAGCAA-3′	5′-CGTTGGCTGCGTTCATGTCGTAAT-3′
TNF-α	5′-CCCACGTCGTAGCAAACCACCA-3′	5′-CCATTGGCCAGGAGGGCGTTG-3′
IL-6	5′-CCTGGAGTTTGTGAAGAACAACT-3′	5′-GGAAGTTGGGGTAGGAAGGA-3′
GAPDH	5′-AGTCTACTGGCGTCTTCA-3′	5′-TTGTCATATTTCTCGTGGT-3′

BMP-2, bone morphogenic protein-2; CGRP, calcitonin gene-related peptide; TRPV1, transient receptor potential vanilloid 1; CXCR4, C-X-C chemokine receptor type 4; MIF, macrophage migration inhibitory factor; TNF-α, tumor necrosis factor-alpha; IL-6, interleukin-6; GAPDH, glyceraldehyde 3-phosphate dehydrogenase.

## Data Availability

The data presented in this study are available on request from the corresponding author.

## Supplementary Materials

Supplementary Materials are available online at https://www.mdpi.com/article/10.3390/ijms22168983/s1.
