# Supplementary material for: Altered Differentiation of Tendon-Derived Stem Cells in Diabetic Conditions Mediated by Macrophage Migration Inhibitory Factor
Source: Int J Mol Sci. 2021 Aug 20;22(16):8983. doi: 10.3390/ijms22168983 (PMC8396498; doi:10.3390/ijms22168983)
Supplement: Supplementary file 1 [file ijms-22-08983-s001.zip › ijms-1328764-supplementary.pdf]

Supplementary Figure

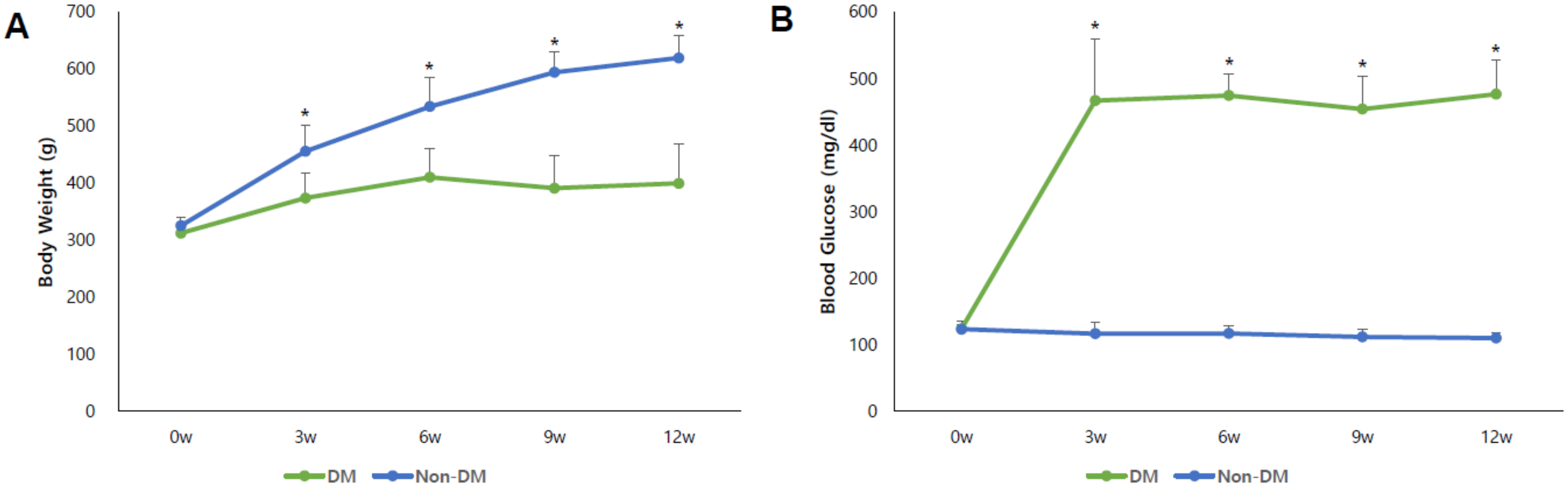

Figure S1. (**A**) Body weight and (**B**) blood glucose level change after streptozotocin or saline treatment in Sprague Dawley rats. Type 1 diabetes was confirmed by continuous high glucose level greater than 270 mg/dL. All values are expressed as mean standard ± deviation. *: statistical significance between diabetic (DM) and non-DM group. None of the rats died or showed any evidence of illness during the study period.
